# Application of Smart Sensors in Commodity Management

**DOI:** 10.3390/s26103096

**Published:** 2026-05-14

**Authors:** Chao-Kong Chung, Meng-Yun Chung, Guo-Ming Sung

**Affiliations:** Department of Electrical Engineering, National Taipei University of Technology, Taipei 10608, Taiwan

**Keywords:** Monitor and Control System (MCS), Network Simulator 2 (NS2), ZigBee, artificial neural, fuzzy control

## Abstract

Integrating sensors with wireless communication capabilities into smart wireless sensing devices allows us to form a wireless sensing network. This network works in conjunction with monitors to display and control parameters at different locations or in the environment. By deploying a wireless sensing network, the system can interact with the user by sending notifications when necessary, based on the environmental conditions and user activities detected by the wireless sensors, and make corresponding adjustments to or control the environment. The advancement and widespread adoption of the internet have enabled the development of this technology. Wireless sensors are widely used in product positioning and environmental monitoring management, making the management of complex products more accurate. The Monitor and Control System (MCS), which combines network cameras and wireless sensors with neural network technology and fuzzy control systems, improves the existing positioning method and enhances positioning accuracy. Product management, which comprises comprehensive digital services and is facing serious staff shortages, has turned to digital payment to reduce labor costs. This experiment was simulated using Network Simulator 2 (NS2). In the sensing system part, the application of a ZigBee network and its status were explored, and interference was analyzed. Information on network interference simulations and their impact on normal services was compiled for network management purposes. Using NS2 network simulation, this study utilizes ZigBee with different neuron nodes and different training times to find the best network model, compares various queuing mechanisms and functions as a network interference intrusion detection system, and explores its node defense capabilities in cases of interference. Node Density: Node density is typically determined by the number of nodes in the simulation area and the size of the scene. Low Density: Sparse node distribution, prone to network partitioning, is suitable for testing latency-tolerant networks (DTNs) or route discovery capabilities. High Density: It entails dense node distribution, severe signal interference, and packet collisions. It is suitable for testing MAC layer collision prevention mechanisms (such as CSMA/CA) and the scalability of outing protocols. Configuration Method: the “set Dest” tool is used in a Tcl script to generate a mobile scene file, defining the number of nodes, range (X, Y), and time to be more significant in product management.

## 1. Introduction

**Figure 1:**
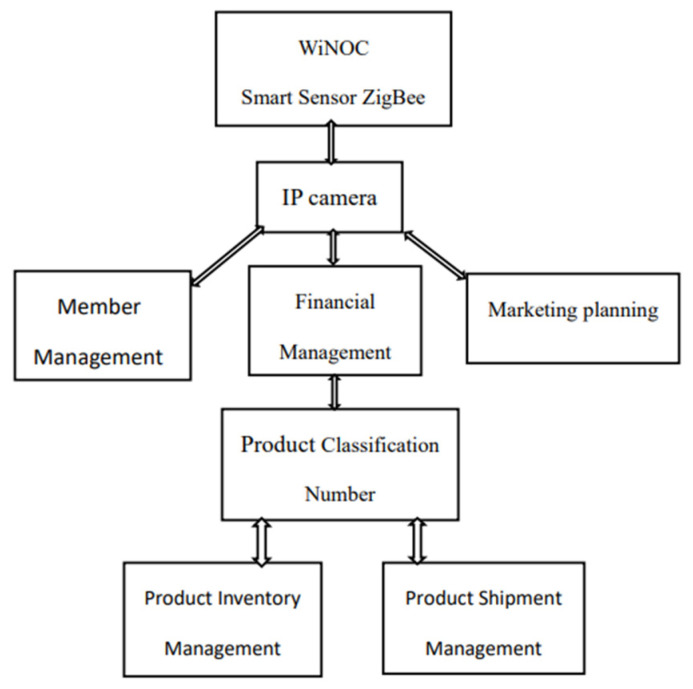
The structure of the shopping mall sensor product inventory management flowchart.

As sensor technology and wireless communication continue to progress, comprehensive digital management can reduce labor costs. During severe staff shortages, it can also reduce operating management costs, prompting the rapid development of wireless sensor network (WSN) applications, as shown in Figure 1. Given that IEEE 802.11 and IEEE 802.15 IEEE 802.11 (Wireless Local Area Network) was proposed/established in 1990. To develop a unified wireless local area network standard, the earliest standard specification was IEEE 802.11-1997, released in 1997.have distinct characteristics and many of their features are complementary, the two are combined in related applications. These two wireless technologies integrate more than one wireless communication technology into smartphones. Therefore, the application of wireless communication has been developing towards the integration of heterogeneous wireless technologies. The related wireless communication technologies and wireless sensor networks enable various real objects to be connected to the Internet, execute specific remote-control programs and achieve the Internet of Things, and can be used in positioning and environmental monitoring. A Monitor and Control System (MCS) combining network cameras and wireless sensors is proposed for warehouse management to improve the existing positioning method and enhance positioning accuracy [1,2,3,4]. As for the sensing device, the Programmable System-on-Chip (PSoC) embedded substrate is used to connect to the temperature and humidity sensing chip [5], and the Universal Asynchronous Receiver/Transmitted (UART) method is used to convert the signal to the ZigBee network module [6], which is then sent to the backend server Wireless Network-on-Chip (WiNOC) system for reception [7,8]. In the case of abnormal conditions and node movement, video cameras are activated for monitoring. The simulation was performed using Network Simulator 2 (NS2). In the sensing system, the ZigBee network and security status were analyzed, and the network sensing simulation was performed [9]. The impact of attacks on normal services was observed, network information was collected, and interactive functions were achieved under intelligent control. The normal packet loss during attacks was simulated using NS2 network simulation to improve node defense capabilities and apply it to commodity and IoT management [10]. Information such as wireless network signal strength can be used to determine the distance between nodes. ZigBee transmits data about each node under test back via the wireless network; then, the server behind it uses triangulation.

## 2. Material and Methods

This study explores and analyzes the application of wireless communication functions and sensors in smart wireless sensing devices, as well as their integration with ZigBee networks and their security. To facilitate network management, this study collects information on network attack simulations and their impact on normal services. Based on this, a wireless sensing network can be constructed. This network works in conjunction with monitors to display and control various parameters in different locations or environments.

**Figure 2:**
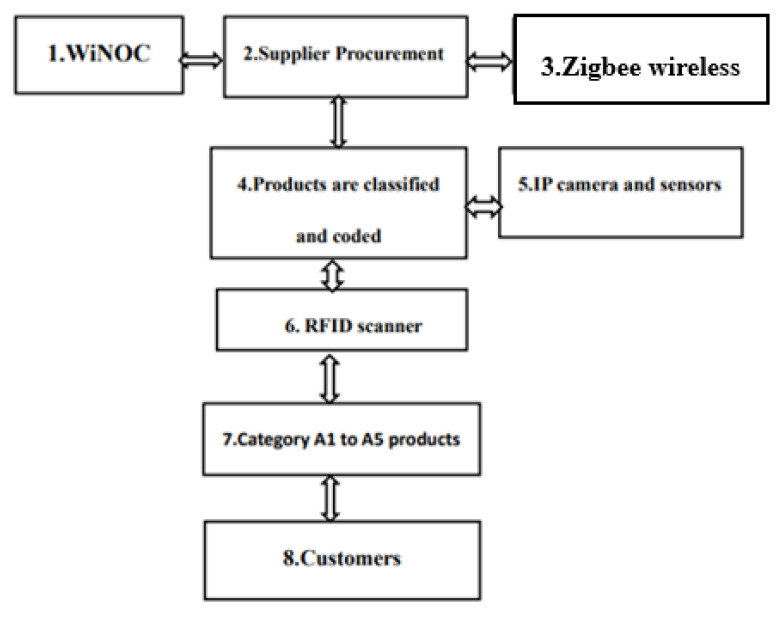
This whole-system work process.

Each step in the process has its own tasks and attributes, making the process smoother, as shown in Figure 2.

The widespread installation of cameras, due to the large amount of data transmission, requires substantial computation and analysis to identify potential unexpected events. The ceiling-mounted cameras are also uniquely shaped and equipped with dual lenses (one above the other) and possess 3D depth sensing capabilities. Furthermore, in the shopping mall whole-system work process, the cameras are positioned at slightly different angles, pointing their lenses at the aisles below or at the goods on the shelves to detect and track the movements of those picking up their orders. Unlike traditional convenience stores, the shelves are tagged with RFID electronic tags, eliminating the need for manual tag replacement [11]. All goods in the logistics center are neatly arranged on the shelves, and each item placed in a perfect order.

The whole-system work process comprises the following:

1. WiNOC: it serves as the system operation and control center.
2. Supplier procurement: products are delivered to the warehouse.
3. Zigbee wireless: a local wireless network.
4. Products are classified and coded: all incoming products are categorized and coded upon entering the warehouse; then, each product is filed separately and added to the computer inventory management system.
5. IP camera and sensor: a photo sensor transmits messages.
6. RFID scanner: a product shipping code scanning and control device.
7. Category A1–A5 products: products are categorized and distributed to different partner manufacturers/customers based on their attributes.
8. Customers: the end user.

**Figure 3:**
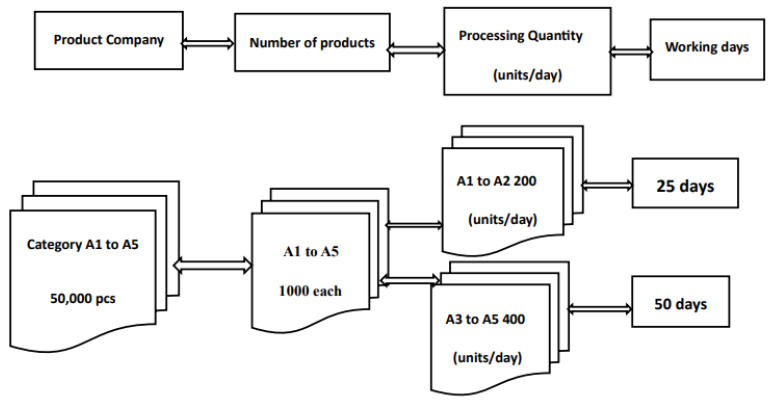
Human management.

**Figure 4:**
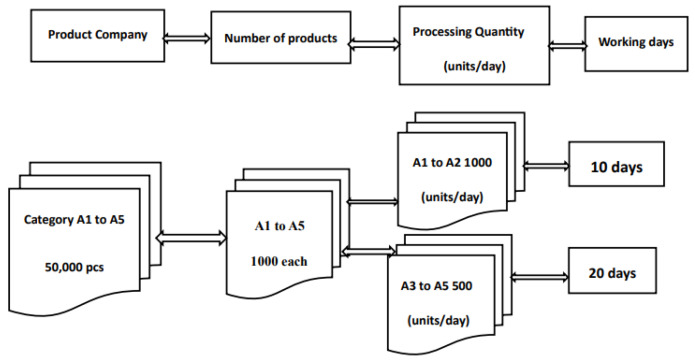
Zigbee digital management.

A comparison of IP cameras and analog cameras, manual processing, and Zigbee digital management as shown in Figure 3 and Figure 4.

### 2.1. Three Main Parts of Product Management

**Figure 5:**
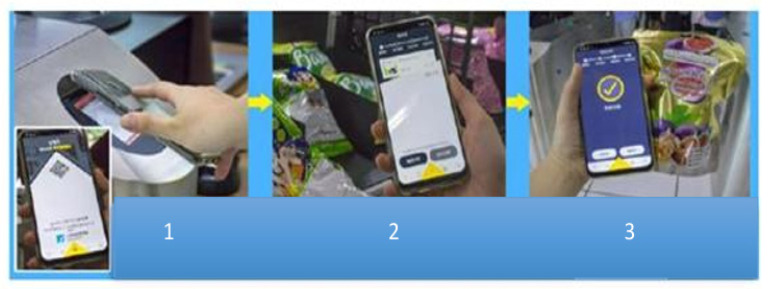
A flowchart of the process of entering a shopping mall.

Before entering the management center, the user must download the app on their smartphone, complete their personal information input, and then go to the front gate control gate website to download the inventory data, as shown in Figure 5 [12].

The process is as follows:

① The user can only enter after linking their mobile app to the payment system.
② The app’s shopping cart instantly displays the items the user has selected.
③ The user can pay directly with the mobile app and skip the queue.

The product supplier provides a designated entry identification code, i.e., a QR code [13]. This code is simultaneously detected by the wireless sensor network detection and sensing and immediately performs the pickup sensing and statistics action. Traditional sensors can collect and report the environmental parameters. However, by integrating wireless communication functions with sensors by deploying smart wireless sensing devices to form a wireless sensing network, which works in conjunction with a monitor, the system can display and control parameters at different locations or in the environment. The system can adjust or control the environment based on the environmental conditions and user activities detected by the wireless sensors and interact with the user by sending notifications when necessary. When the manufacturer confirms the completion, the system deducts the amount from the credit card/passbook number linked to the relevant financial institution. After shopping, a user can scan the QR code at the exit, enter their information, and complete the payment process. The exit door opens automatically when the user leaves, and they can take their goods without swiping their card.

### 2.2. Merchandise Area

**Figure 6:**
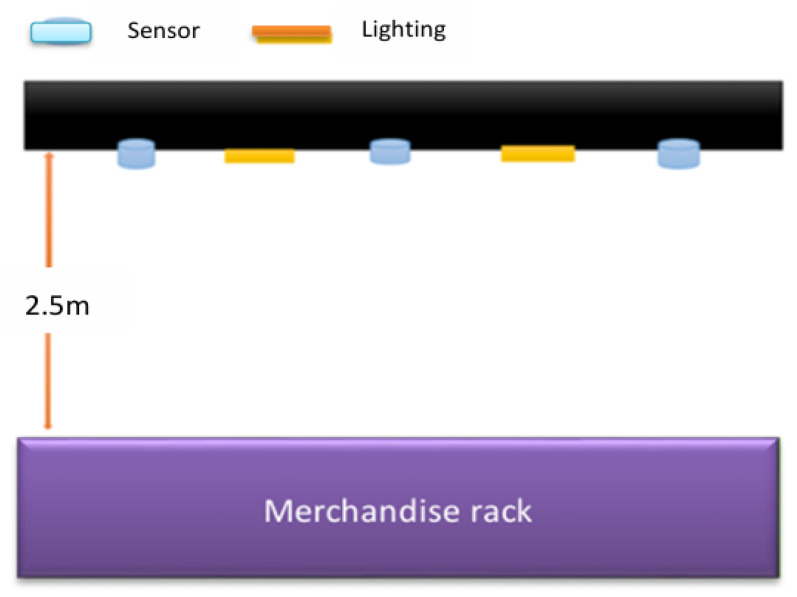
A roof-mounted array sensor used for product management.

A roof-mounted array wireless sensor network is used for product management. Customers walk into the store through the gate and see a variety of merchandise on the shelves, and they can just take what they want and go. This immediately solves the problem of too many wires. Wireless sensor modules can be quickly adapted to integrate various sensor devices, as needed, and these sensor modules can be used for temperature detection, humidity monitoring, light monitoring, etc. Through wireless sensing positioning technology, the relevant environmental factors are transmitted back to the backend server, as shown in Figure 6.

With the rapid development of wireless network technologies such as RFID, ZigBee, Bluetooth, and Wi-Fi, various corresponding sensing modules and sensing devices have also emerged, together forming wireless sensor networks (WSNs) for monitoring environmental information, as described in Table 1 [14,15,16,17]. Zigbee is a low-power, low-data-rate, short-range wireless mesh communication technology based on the IEEE 802.15.4 standard, widely used in smart homes, industrial automation, and the Internet of Things (IoT). It supports multi-node mesh structures, is suitable for battery-powered devices, and offers high reliability and security. The backend server uses positioning methods such as triangulation and sampling analysis to calculate node coordinates. By transmitting signal strength and other information via wireless network, the distance between nodes can be determined, and the data can be sent back to the server. The error value is reduced through subsequent judgments such as the trajectory method and the path method, and the positioning factors and radio wave interference caused by obstacles that affect the positioning system are improved. Artificial intelligence-based fuzzy control systems improve positioning accuracy and effectively reduce errors, and they are used as training modules for sample analysis [18]. For the different hidden layer neuron nodes of the entire commodity logistics, they are used for network sensing, intrusion detection, control, and identification. Comparing various queuing mechanisms and their functions, using NS2 network simulation, the normal packet loss during an interference, and the defense capabilities of different queue management mechanisms against blocking service interference, whenever an anomaly occurs, the system center immediately sends out an anomaly signal and also displays the location and cause of the incident. Staff then immediately take steps to remove the obstruction. In fact, the radio wave interference caused by obstacles still affects the accuracy of the positioning system. Therefore, to improve positioning accuracy and effectively reduce errors, the complete inbound and outbound management of commodity inventory is applied [19].

**Figure 7:**
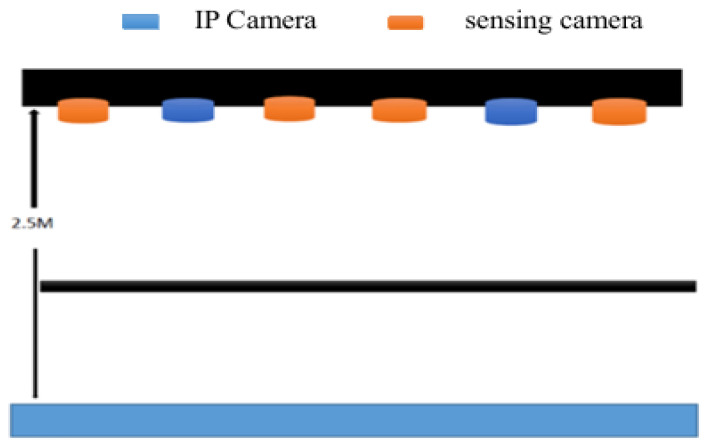
An IP camera array composed of sensing cameras used for product management.

**Figure 8:**
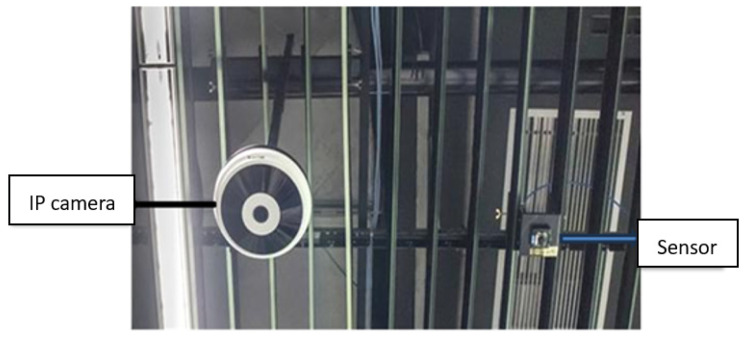
A high-sensing camera array for unmanned stores (real-world photos).

In the logistics center, the IP camera and sensing camera array consisting of cameras that sense a depth of more than 2.5 m is mainly used to combine computer vision and image recognition systems, as shown in Figure 7 and Figure 8 [20]. By analyzing various picking actions, it can automatically detect product categories and track the movement path in the warehouse to determine what kind of products the picker has taken. Cameras that are suspended from the ceiling target goods in the aisles or on shelves below from different angles to detect and track every movement of the person picking up their goods. It uses image recognition technology to detect the movement of goods and automatically determine the type of goods taken [21]. Previous monitoring systems could only perform timed monitoring and could only monitor specific locations. They were unable to monitor the current situation of the monitor in a timely manner. There were considerable gray areas, and they were unable to conduct comprehensive monitoring. Consequently, when emergencies occurred, they were unable to effectively supervise and control, resulting in many flaws. Moreover, widely installing cameras requires significant computing and analysis due to the large amount of transmitted data [22].

The smart logistics center has created a full-scale image recognition system. The camera is installed in a fixed box and hung just below the ceiling with exposed steel frames and pipelines. It can be seen as long as the customer looks up. The shape of the camera on the ceiling is also different. The camera is equipped with two lenses, one above and one below, and it has 3D height sensing capabilities [23]. In addition, the camera is positioned at slightly different angles, with the lens aimed at the aisle below or the goods on the shelves, to detect and track the actions of the pickers. Unlike typical convenience stores, the shelves here are equipped with RFID tags, i.e., electronic tags, which do not require manual label replacement [24]. All merchandise in the logistics center is neatly placed on the shelves. In addition, in the temperature and humidity sensing part, the Fontal FT-6200 ZigBee temperature and humidity sensing module is used for monitoring fresh food products that are usually easy to identify but become difficult to identify when placed in the same outer packaging; thus, a special identification design is added to their packaging, allowing the camera on the ceiling to more easily identify what kind of food the customer has taken. Multi-function sensors are integrated on the shelves as an aid to compare the results of image recognition, thus improving recognition accuracy. The data collected by these sensors is also analyzed to improve staff efficiency [25]. Using Cypress’s Psoc CY8C29466 chip, the RF interface is transferred to the ZigBee network module and sent back to the backend server. For example, when a product is about to be sold out, a message is automatically sent to notify the clerk to restock it. If a product is misplaced or placed on the wrong shelf, a warning is issued. The clerk can check it from a handheld device and put the product back on the correct shelf. Its outstanding ability in identifying products and analyzing customer behavior can even be used to prevent theft. Even if consumers try to deceive it, it is difficult to escape its eyes. It is even smarter than the store clerks and has no blind spots. With a large number of cameras and sensors present in the store, it can track the products that managers take off or put back on the shelves, and when the products on the shelves are deliberately covered with shopping bags, it can still successfully identify and check the product details of the covered items.

### 2.3. Payment

Advances in wireless sensor networks have reduced labor costs through digital payments. Customers automatically receive their bill details when they leave the store. After picking up their purchases and leaving, customers receive a mobile bill notification, allowing them to view their shopping details directly on their phones. The transaction is then deducted from their designated bank account to settle the payment. In this AI-powered, fully digital payment service, all purchases made in the shopping mall are automatically deducted from a designated credit card or bank account, thus completing the settlement of the transaction. The AI-powered, fully digital payment service also explores how organizations can use innovative methods and organizational reengineering to revitalize the company and pursue higher sales targets.

## 3. Results and Discussion

### 3.1. The Equipment

**Figure 9:**
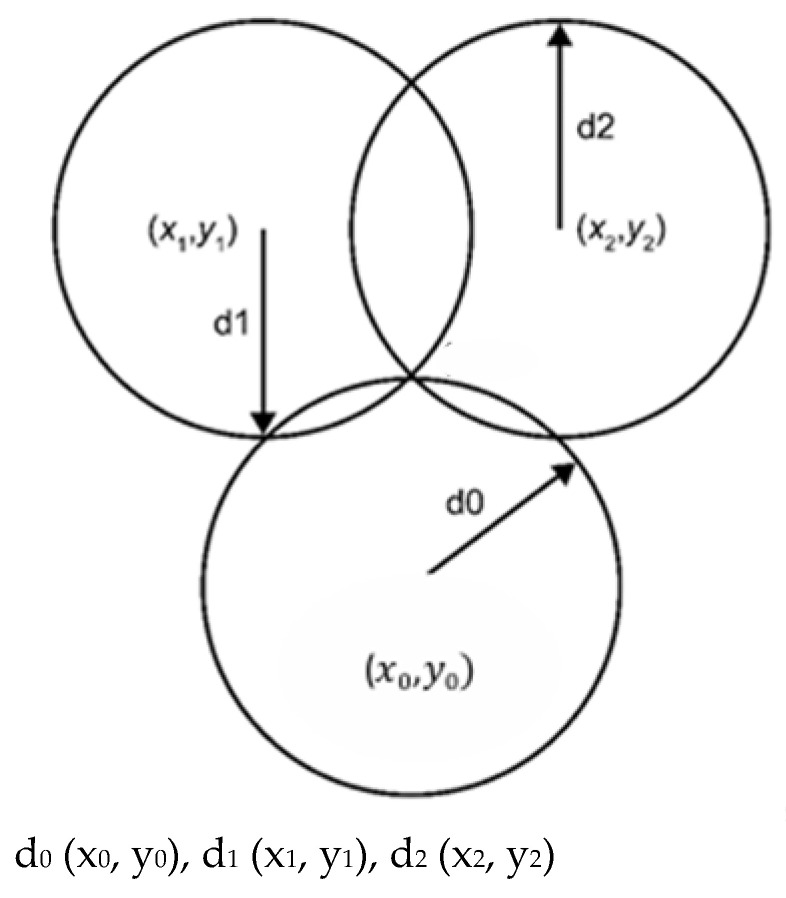
Three-point localization method for wireless sensor networks.

In the positioning mechanism of the wireless sensor network, due to the errors caused by the signal transmission time and the logarithmic attenuation of the signal with respect to the distance, the application of ZigBee introduces an artificial intelligence system to improve the accuracy of positioning through fuzzy control, and neural fuzzy systems typically consist of five layers: input fuzzification, rule inference, normalization, defuzzification (consequent layer), and total output. This allows the system to automatically correct for precise boundaries in the data that are difficult for humans to define, thereby improving the accuracy of the positioning system and effectively adapting to the problem that the location of the wireless sensor network nodes may move frequently, making the monitoring effect more accurate. The large amount of data generated during the wireless transmission process is effectively trained through the section that addresses various issues that may arise during the entire product management data transmission process. Neural networks are utilized to filter and check input concealment before finalizing the output, and fuzzy control is used to improve positioning accuracy and reduce error. The ZigBee algorithm transmits data from the node under test back to the wireless network. Sampling and analysis methods are used to calculate the node coordinates; then, the backend server uses triangulation to reduce the error value and to improve positioning accuracy, as shown in Figure 9. In fact, the radio wave interference caused by obstacles still affects the accuracy of the positioning system. Therefore, to improve positioning accuracy and effectively reduce errors, a fuzzy control system using artificial intelligence was used as a training module for sample analysis. By using information such as wireless network transmission and signal strength, the distance between nodes can be determined, and the data is transmitted back. The backend server uses positioning methods such as triangulation and sample analysis to calculate the node coordinates. Subsequent judgments such as the trajectory method and path method are used to reduce the error value and improve the positioning system by addressing the impact of radio wave interference caused by factors and obstacles.

Trilateration is a commonly used positioning algorithm involving the following:

The positions of three points: (x_0_, y_0_), (x_1_, y_1_), and (x_2_, y_2_);

The known unknown points: (x_0_, y_0_);

The distance to three points: d_0_, d_1_, and d_2_.

We draw three circles with radii d_0_, d_1_, and d_2_. Using the Pythagorean theorem, we derive the formula for calculating the position of the intersection point, i.e., the unknown point:

(x − x_0_)^2^ + (y − y_0_)^2^ = d_0_^2^ (1)

(x − x_1_)^2^ + (y − y_1_)^2^ = d_1_^2^ (2)

(x − x_2_)^2^ + (y − y_2_)^2^ = d_2_^2^ (3)

Furthermore, to improve positioning accuracy and effectively reduce errors, an AI-driven fuzzy control system is used as a training module for sample analysis. Throughout the product logistics process, different hidden layer neural network nodes are used for network perception, intrusion detection, control, and identification. Various queueing mechanisms and functions are compared, and using NS2 network simulation, the system demonstrates the defense capabilities against blocking-service attacks by employing different queueing management mechanisms in the event of normal packet loss during an attack. This provides comprehensive inbound and outbound management for the product inventory.

This study uses a smart sensor combined with a ZigBee wireless sensor network for environmental monitoring, as described in Table 2. In the event of an anomaly, the data is transmitted back to the backend server via the network, which then activates nearby cameras for monitoring and recording. ZigBee applications integrate artificial intelligence systems, enabling remote personnel to clearly observe abnormal situations through nearby cameras. In the positioning mechanism of wireless sensor networks, errors occur due to signal transmission time and the logarithmic attenuation of signal strength with distance. Through fuzzy control, the accuracy of positioning is improved, enhancing the overall accuracy of the positioning system. This also effectively addresses the issue of frequent movement of wireless sensor network nodes, resulting in more precise monitoring. In warehouse management, wireless sensor networks are used, and the settings of each wireless node can be quickly adjusted and changed according to system requirements. The wireless sensor module can integrate various sensing devices as needed, such as temperature, humidity, and light intensity monitoring. Through wireless sensing positioning technology, relevant environmental factors are transmitted back to the backend server. After passing through the gate, customers enter the store and find various goods displayed on the shelves for them to grab and leave.

It has built-in computer processing capabilities and can transmit real-time audio and video via wired or wireless networks (Wi-Fi), allowing users to remotely monitor and record using devices such as mobile phones and computers. A digital camera is a photographic device that uses a network to transmit digital images. It provides a smarter and more flexible monitoring solution than traditional cameras, supporting functions such as high resolution, motion detection, and AI analysis, as described in Table 3.

Shopping mall management comparison:

A comparison of IP cameras and analog cameras, manual processing, and Zigbee digital management are described in Table 4 and shown in Figure 2, Figure 3 and Figure 4. The products A1 to A2 are relatively light, so there are more of them when sorting, while products A3 to A5 are relatively heavy, and there are fewer of them when sorting. If a company plans to receive 50,000 units of five different types of products with varying weights into its warehouse, the inventory management practices are as follows:

1. The human resources-based management approach:

The 15 employees are divided into five groups of three, each responsible for products A1 to A5. The products are sorted, packaged, and numbered manually. Each group of A1 to A2 products processes 400 units per day, which takes 25 working days to complete, based on an 8 h workday. Then, the different product categories are categorized and managed. Each group of A3 to A5 products processes 200 units per day, which takes 50 working days, based on an 8 h workday.

2. Zigbee digital management approach:

The 15 employees are divided into five groups of three, each responsible for products A1 to A5. The products are sorted, packaged, and numbered manually. Each group of A1 to A2 products processes 1000 units per day, which takes 10 working days to complete, based on an 8 h workday. Then, the different product categories are categorized and managed. Each group of A3 to A5 products processes 500 units per day, which takes 20 working days, based on an 8 h workday. The Zigbee network system utilizes intelligent sensors and photography-related equipment, with manpower as a foundation, to improve management efficiency.

### 3.2. Discussion

The relevant key research highlights and applications are as follows: Optimized Model Design: adjusting the backpropagation neural network structure (different neuron nodes) and training time to improve the accuracy of anomaly signal identification. Security Defense Mechanisms: utilizing NS2 to simulate environments under specific network attacks and comparing the advantages and disadvantages of different queuing mechanisms (such as FIFO and PQ) in terms of node defense capabilities and traffic handling. Product Management Applications: experimental results can be imported into actual smart sensors or ICT products to achieve real-time intrusion detection and improve overall environmental quality and network security.

The application of wireless sensors in product positioning, environmental monitoring and management, etc. makes product management more accurate. The Monitor and Control System (MCS) using network cameras and wireless sensors, combined with neural network technology and fuzzy control system, has improved the existing positioning method and achieved positioning accuracy. The results of this study indicate that processing 50,000 products by normal personnel takes a relatively long time. In contrast, under Zigbee digital management, the product processing time of A1 to A2 is reduced by 60% and A3 to A5 is reduced by 50%, making it the most efficient and cost-effective approach for enterprise management.

## 4. Conclusions

To effectively monitor environmental factors, the environment is monitored through corresponding sensing modules based on common environmental factors. Illumination, ultraviolet rays, and other parts are monitored using ZigBee network positioning and sensing modules. Lighting, ultraviolet radiation, and other components are monitored using ZigBee network positioning and sensing modules. Finally, through the PSoC embedded system control board, the RF interface is transferred to the ZigBee network module and sent back to the backend server. After the test is completed, data recording can begin. When abnormal data is generated and exceeds the warning threshold, the camera is immediately activated. The system automatically compares the abnormal data with the warning threshold, transmits the image location data to a remote server, and sends the data to maintenance personnel for review via SMS. Compared to traditional camera monitoring systems, this system can capture abnormal situations in real time and send signals to notify external monitoring personnel. It also verifies that the ZigBee network can effectively transmit data even in an environment with mobile nodes and can quickly transmit data and achieve results. The core difference in the application of sensors in product inbound and outbound processes lies in the shift from manual, inefficient “passive recording” to automatic, real-time, and high-precision “active monitoring and data management.” Traditional management relies on the manual verification of documents, which is prone to errors, while sensors (such as photoelectric and RFID) can automatically detect, identify, and count, enabling unmanned automated inventory, providing real-time inventory and dynamic information, greatly improving efficiency and accuracy, and reducing costs, thus achieving intelligent warehousing and supply chain management.

With advancements in sensor technology and wireless communication, their application in merchandise management is becoming increasingly sophisticated. Combined with technologies such as AI and IoT, and with the continuous innovation of business models, setting up a store requires the consideration of various factors. Smart sensor technology has emerged to address this need in merchandise management. However, managing the products sold in unmanned stores presents a significant challenge. The timely development of artificial intelligence (AI) has led to the transformation of traditional coin-operated vending machines into digital contactless payment systems. AI-powered, fully digital payment management services are being implemented in multi-functional stores offering a variety of products where processing 50,000 products by normal personnel takes a relatively long time. In contrast, under Zigbee digital management, the product processing time of A1 to A2 is reduced by 60% and A3 to A5 is reduced by 50%, making it the most efficient and cost-effective approach for enterprise management. Digital payment systems can reduce labor costs and operational management costs.

## Figures and Tables

**Table 1:** A comparison of indoor network transmission equipment.

Title	Wi-Fi	ZigBee	Power Carrier	Bluetooth
Distance	100–300 m	50–300 m	500 m	1–10 m
Current	10–50 mA	5 mA	Low-power AM radio signals	Between ZigBee and Wi-Fi
Application surface	Widely	Low power consumption, mesh networking, high reliability, security, versatility, etc.	Can be transmitted based on power lines without wiring	Refers to the user interface (UI) or settings area on a device

**Table 2:** A comparison of various monitoring systems.

Monitoring System	Human Monitoring	GPS Positioning	This Study
Operation time	Long	Short	Short
Human resources consumption	Many	Middle	Few
Monitoring time	Extremely short	Middle	Long
Positioning accuracy	Middle	Short	High

**Table 3:** A comparison of digital cameras (network digital cameras) and traditional cameras.

Dimension	Digital Camera	Traditional Camera
Image transmission	Transmitting images via the Internet	Transmission via analog signal
Construction wiring	A single network cable is sufficient to transmit both signal and power, allowing a single line to be used to connect back to the host computer via a router.	Signal cables and power cables are required, and each camera needs to be connected back to the main unit, requiring a large number of cables.
Construction price	High-cost, expensive equipment	Low-cost, affordable equipment
Image signal	Through digitized signals	Through analog signals
Applicable environment	Suitable for large-scale applications	Suitable for small-to-medium-sized areas
Function	It has multiple functions, such as transmitting images, voice, air conditioning relative humidity, and other relevant information to the host via Zigbee network for easy management.	Its function is simple, except for transmitting video images back to the monitoring host system.

**Table 4:** A comparison of product handling methods.

Category 50,000 pcs	Quantity (Pieces)	Human Management			Zigbee Digital Management		
		Manpower	Processing Quantity (Units/Day)	Working Days	Manpower	Processing Quantity (Units/Day)	Working Days
A1	10,000 each	3	400	25	2	1000	10
A2		3	400	25	2	1000	10
A3		3	200	50	2	500	20
A4		3	200	50	2	500	20
A5		3	200	50	2	500	20

## Data Availability

The data used to support the findings of this study are included within the article.
